# Evaluation of extrapancreatic inflammation on abdominal computed tomography as an early predictor of organ failure in acute pancreatitis as defined by the revised Atlanta classification

**DOI:** 10.1097/MD.0000000000006517

**Published:** 2017-04-14

**Authors:** Chenyang Chen, Zixing Huang, Hang Li, Bin Song, Fang Yuan

**Affiliations:** Department of Radiology, West China Hospital, Sichuan University, Chengdu, China.

**Keywords:** acute pancreatitis, computed tomography, extrapancreatic inflammation, organ failure, revised Atlanta classification

## Abstract

The aim of the study was to determine whether extrapancreatic inflammation on computed tomography (EPIC) is helpful in predicting organ failure in the early phase of acute pancreatitis (AP) as defined by the 2012 revised Atlanta classification.

Patients (n = 208) who underwent abdominal computed tomography (CT) within 24 hours after AP onset and admission were retrospectively identified. Each patient's EPIC score, Balthazar score, bedside index of severity in acute pancreatitis (BISAP), and systemic inflammatory response syndrome (SIRS) score were obtained. Primary endpoints were organ failure occurrence and death. Scores were evaluated by receiver operator characteristic (ROC) curve and area under the curve (AUC) analysis.

Median age was 45 years (range: 18–83 years). Forty-seven patients (22.6%) developed organ failure, and 5 patients (2.4%) developed infection and underwent surgery. Two patients died. The median EPIC score was 2 (range: 0–7). EPIC score accuracy (AUC = 0.724) in predicting organ failure was similar to that of BISAP (0.773) and SIRS (0.801) scores, whereas Balthazar scoring was not significant (*P* = .293). An EPIC score of 3 or greater had a sensitivity and specificity of 80.65% and 63.16%, respectively. EPIC scores correlated moderately with organ failure severity (Spearman *r* = 0.321) and number of failed organs (*r* = 0.343).

The EPIC scoring system can be useful in predicting the occurrence of organ failure, but it does not differentiate severity and number of failed organs in early phase AP.

## 1 Introduction

Acute pancreatitis (AP) is a dynamic process with 2 overlapping phases of disease: the early phase and the following late phase.^[1]^ The early phase usually lasts for 1 week, and the late phase lasts for weeks to months. In the early phase, the severity of AP primarily depends on the presence and duration of organ failure due to a systemic inflammatory response that is not necessarily correlated with the infection or the extent of necrosis.^[2,3]^ Thus, prediction of organ failure in the early phase is very important to evaluate the risk stratification of AP.^[4,5]^ Clinically, acute physiology and chronic health examination II (APACHE II) and Ranson scores have been commonly used to assess and predict the severity of AP. In addition, serum levels of the inflammatory mediators interleukin (IL) 6 and IL-10 proved to be accurate for predicting organ dysfunction in AP patients,^[6–8]^ an increased neutrophil to lymphocyte ratio^[9,10]^ and an decreased of peripheral blood CD4+ T cell^[11]^ considered to be an independent risk for persisitent organ failure. However, these indicators have not been widely used in clinical practice because of difficulty in calculating of APACHE II scores and the short half-lives of IL-6 and IL-10.

In addition to assessment of the aforementioned relevant clinical and biochemical parameters in AP, computed tomography (CT) scanning is considered the reference standard not only for diagnostic purposes but also for assessing the severity of AP.^[12,13]^ Enhanced CT severity index (CTSI) and unenhanced Balthazar score have been regarded as the conventional CT scoring systems. However, some investigators have suggested that CT on admission is not useful because it takes a few days for pancreatic necrosis to develop, and CT scans obtained in the early phase of AP would not change clinical management.^[14–16]^ They also have recommended that CT studies should be reserved for only those patients predicted to have severe AP by clinical assessment. Moreover, the use of contrast agents may worsen the course of the pancreatitis.^[17]^

Other investigators^[13]^ have evaluated the utility of extrapancreatic changes as a diagnostic tool for early phase AP. Schroder et al^[18]^ have reported that extrapancreatic changes were more frequent in patients with hemorrhagic pancreatitis. Hjelmqvist et al^[19]^ reported similar findings, and they suggested that peripancreatic edema, retroperitoneal and intraperitoneal edema, bowel distension, and pleural effusion should be incorporated into the scoring system. However, the prognostic value of this score using receiver operator characteristic (ROC) curve analysis was never determined. In 2004, Mortele et al^[20]^ used a modified CTSI which included extrapancreatic findings such as pleural effusion, ascites, vascular or parenchymal complications, and gastrointestinal tract involvement. They found that this scoring system correlated with outcome parameters better than the commonly used CTSI. These studies indicated that extrapancreatic inflammation plays an important role in predicting the severity of AP. In 2007, De Waele et al^[21]^ developed a new unenhanced CT scoring system called the extrapancreatic inflammation on CT (EPIC) score, and they concluded that this scoring system allowed accurate estimation of disease severity and mortality within 24 hours of admission. They also found that an EPIC score of 4 or higher had an area under the curve (AUC) of 0.91 and had a sensitivity and specificity for predicting severe pancreatitis of 100% and 70.8%, respectively. However, they used the old 1992 Atlanta classification of AP that defined 2 degrees of severity: severe AP and mortality.^[1,22]^ According to the 2012 revised Atlanta classification of AP,^[2]^ 3 degrees of severity are defined: mild acute pancreatitis (absence of organ failure), moderately severe acute pancreatitis (presence of transient organ failure), and severe acute pancreatitis (presence of persistent organ failure). The purpose of this study was to evaluate the utility of the EPIC score for predicting organ failure in the early phase of AP as defined by the revised Atlanta classification.

## 2 Materials and methods

### 2.1 Patients

The independent Ethics Committee at Sichuan University affirmed that neither an ethics committee approval nor patient consent was necessary for this retrospective study. We conducted a retrospective case series study of all adult patients (age > 18 years) who were admitted to West China Hospital (the second most popular class 3A hospital located in Chengdu Sichuan province of China) with a confirmed diagnosis of AP, between December 2015 and June 2016.

Patients were identified according to the International Classification of Diseases, Ninth Revision, Clinical Modification Code for AP (577.0). AP was diagnosed if 2 or more of the following findings were present: characteristic abdominal pain; serum amylase or lipase level 3 or more times higher than the upper limit of normal (i.e., > 210 U/L and 180 U/L, respectively); and an imaging study (CT, magnetic resonance imaging [MRI], or transabdominal sonography) showing changes consistent with acute pancreatitis. Patients receiving an abdominal CT scan within 24 hours after AP onset and admission were considered eligible for the study. The onset of AP was defined as the time of abdominal pain onset.^[2]^ After the etiology and risk stratification of patients were confirmed, appropriate treatment and nursing were implemented, such as prevention and treatment of shock, improving microcirculation, spasmolysis, acetanilide, inhibiting pancreatic enzyme secretion, anti-infection measures, nutritional support, prevention of complications, intensive care measures, and so on. Patients with gallstone pancreatitis were treated with endoscopic retrograde cholangiopancreatography (ERCP), endoscopic sphincterotomy (EST), or cholecystectomy. After CT, ultrasound- or endoscopic ultrasound- guided puncture drainage was implemented, and patients suspected of infection underwent surgery in addition to drainage.

**Figure 1:**
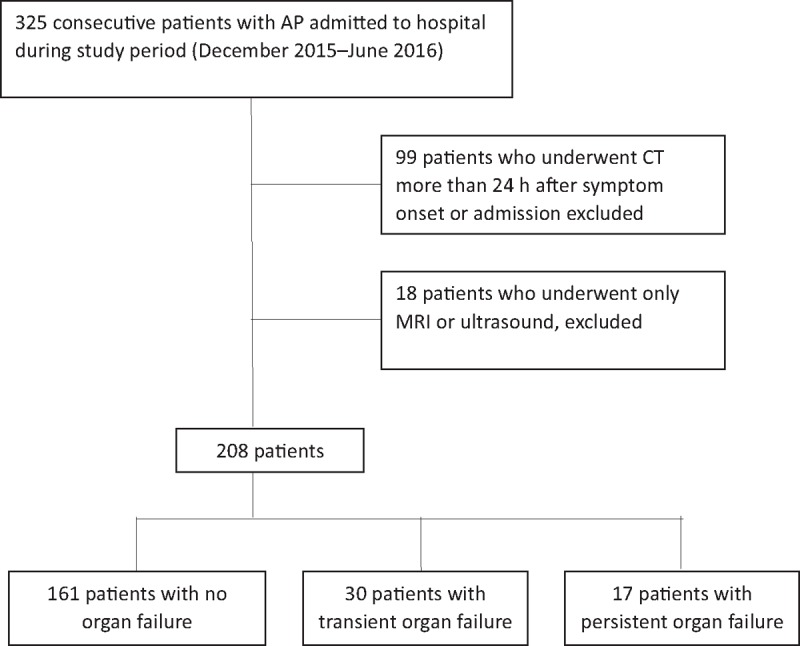
Flow diagram showing the cohort selection process and final diagnoses. AP = acute pancreatitis, MRI = magnetic resonance imaging.

Exclusion criteria were AP in pregnancy, malignancy, traumatic AP, and acute-on-chronic pancreatitis. Three hundred and twenty-five AP patients who were referred to CT on admission at our hospital were identified by the hospital information system. Of these 325 patients, 117 were excluded for the following reasons: 99 received CT examination longer than 24 hours after AP onset or admission, 8 underwent only MRI examination and 10 had only an ultrasound examination. Consequently, 208 patients with available imaging and clinical data were included in this study (Fig. 1). Of these patients, 166 had an unenhanced CT scan and 42 had the enhanced CT scan. One hundred and twenty-one of the enrolled patients were male (58.2%), 87 were female (41.8%), and the median age was 45 years (range: 18–83 years).

### 2.2 Laboratory and clinical data

Baseline data collected included APACHE II and bedside index for severity in AP (BISAP) within 24 hours of admission to our hospital. In addition, the occurrence of systemic inflammatory response syndrome (SIRS) was also recorded within the first week after admission. According to the Marshall multiple organ dysfunction scoring system,^[23]^ organ failure was defined as a score of ≥ 2 in 1 or more of 3 organs (respiratory, renal, and cardiovascular). The duration of organ failure included transient organ failure (≤ 48 hours) and persistent organ failure (> 48 hours).^[16]^

### 2.3 CT technique and imaging data

All examinations were performed on multiple detector CT scanners (Somatom Definition AS 128 and Somatom Definition Flash 128, Siemens AG, Wittelsbacherplatz 2, DE-80333 Muenchen Forchheim, Germany) within 24 h after AP onset and admission. The following standard CT protocol for abdominal imaging was applied: For opacification of the gastrointestinal tract, 2000 mL of positive radiocontrast agent was administered orally, and 150 mL nonionic contrast medium (300 mg I/mL iopromide [Ultravist, Bayer Schering Pharma AG,Berlin Germany] or iomeprol [Iomeron, Bracco Altana Pharma, Milano, Italy]) was power-injected IV at a rate of 3 mL/s in all patients. Portal venous phase scans of the abdomen in the craniocaudal direction were acquired and reconstructed in the axial plane with a slice thickness of 5 mm.

All CT scan data generated at our institution were reviewed independently at workstations loaded with picture archiving communication system software (Syngo-Imaging, version VB36A, Siemens Medical Solutions).

Areas of pancreatic parenchyma that exhibited nonenhancement on contrast-enhanced CT (CECT) were regarded as necrosis.^[2]^ Pancreatic parenchymal necrosis was diagnosed only if a follow-up CECT examination was performed in patients who had previously undergone a plain CT scan. Two radiologists (the first author and the corresponding author) with more than 3 years of experience in abdominal radiology independently reviewed all CT images without knowledge of the patient characteristics and clinical outcome. When the CT findings observed by both radiologists were in agreement, those findings were regarded as the final result. When the CT findings observed by both readers were inconsistent, the 2 radiologists reanalyzed the CT images and a definitive result was reached by consensus. Based on the CT image analysis, the EPIC (Table 1) and Balthazar (Table 2) scores were calculated and recorded for each patient. We did not evaluate the CTSI because only 42 patients in our retrospective cohort underwent CECT.

**Table 1:**
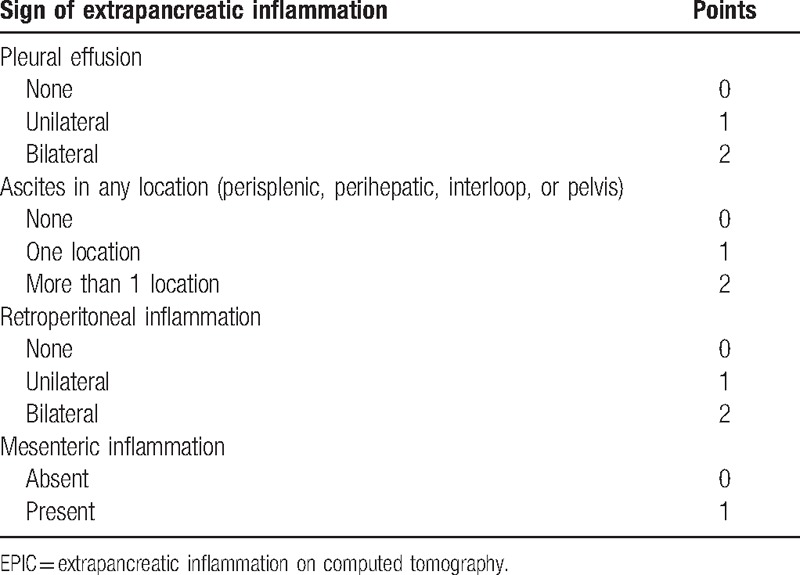
Components of the EPIC score.

**Table 2:**
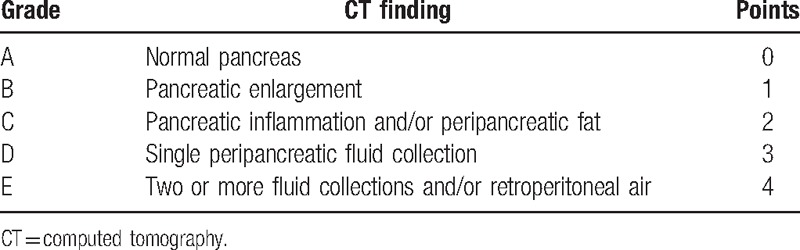
Components of the Balthazar score.

### 2.4 Outcome parameters

The primary endpoints were the occurrence of organ failure and death. Secondary end points were pancreatic or peripancreatic infection (diagnosed by intervention or fine-needle aspiration), need for intervention or operation, and duration of hospital stay. Pancreatic or peripancreatic infection was defined as the presence of Gram stain- or culture-positive fluid obtained by intervention or fine-needle aspiration. Interventions were defined as percutaneous or endoscopic drainage or percutaneous, endoscopic, or surgical necrosectomy.

### 2.5 Statistical analysis

Quantitative results were expressed as either the median or the mean ± standard deviation (SD). All statistical analyses were performed in the SPSS software package (version 19.0, IBM Corporation, Armonk, NY), with the exception of ROC curve analyses that were performed in the MedCalc software package (version 11.4.2.0, MedCalc Software bvba, Belgium). Continuous variables were compared with Student's *t* tests or Mann–Whitney *U* tests. Categorical variables were compared with Spearman rank correlation tests. ROC curves were plotted for EPIC, Balthazar, BISAP, and SIRS scores to assess their ability to predict organ failure. The AUC and 95% confidence interval (CI) for each of these scoring systems was calculated, and the AUCs were compared using *z* tests. An AUC value of 0.50 to 0.69 was defined as low accuracy, 0.70 to 0.90 was defined as moderate accuracy, and > 0.90 was defined as good accuracy. Differences with a *P* value < 0.05 were considered statistically significant. Correlational analyses between EPIC scores and severity of organ failure, between EPIC score and number of failed organs, and between EPIC score and outcome parameters were performed with R × C contingency table tests (McNemar test and Kappa test) and Spearman analyses. A Spearman correlation coefficient with an absolute value in the range of 0.090 to 0.099 was defined as indicating no correlation, those in the range of 0.10 to 0.29 were defined as indicating a weak correlation, those in the range of 0.30 to 0.49 were defined as indicating a moderate correlation, and those in the 0.50 to 1.0 range were defined as indicating a strong correlation.

## 3 Results

### 3.1 Patient characteristics

Clinical characteristics of the entire cohort, patients with organ failure, and patients without organ failure are shown in Table 3. The mean age of all enrolled patients was 47.5 ± 14.3 years. Etiological classifications of pancreatitis observed among these 208 patients included biliary tract stones (n = 97), hyperlipidemia (n = 52), alcohol abuse (n = 30), and idiopathic acute pancreatitis (IAP; n = 29), where the IAP classification was used to describe pancreatitis without an identifiable cause. None etiological classification (biliary tract stones, Spearman *r*_208_ = 0.059, *P* = .451; hyperlipidemia, Spearman *r*_208_ = -0.146, *P* = .061; alcohol abuse, Spearman *r*_208_ = 0.060, *P* = .446; and IAP, Spearman *r*_208_ = 0.045, *P* = .567) differed between patients with versus without organ failure.

**Table 3:**
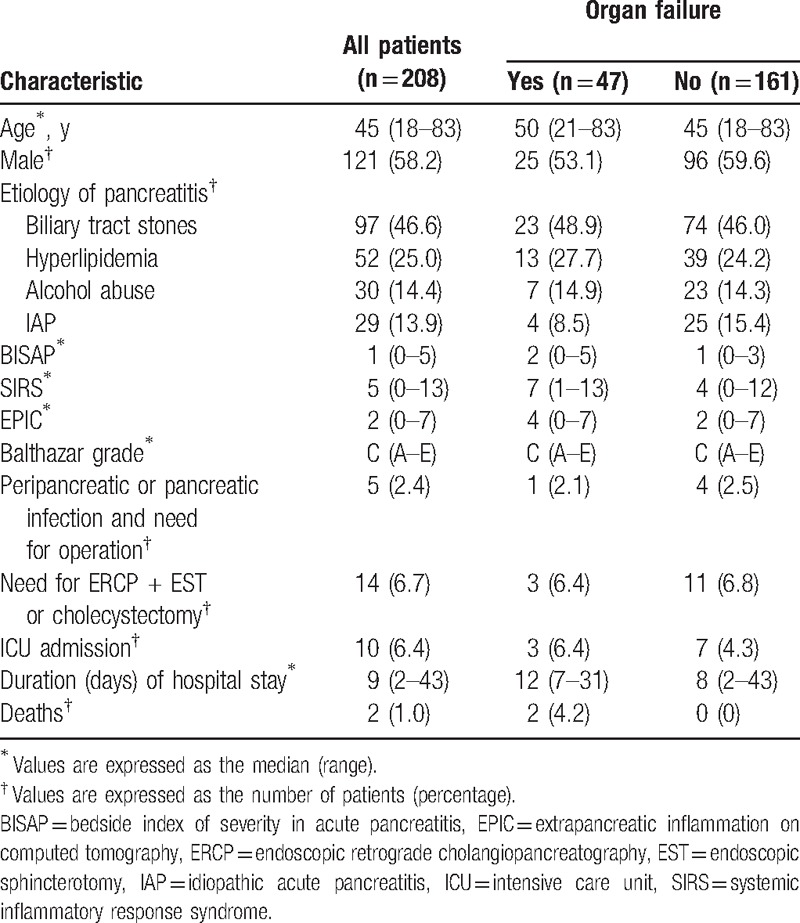
Characteristics of entire cohort and patients with and without organ failure.

Organ failure was present in 47 patients, of which 30 patients had transient organ failure and 17 patients had persistent organ failure. Respiratory failure was observed in 41 patients, of which 34 and 7 had transient and persistent organ failure, respectively. Renal failure was present in 6 patients, of which 5 and 1 had transient and persistent organ failure, respectively. No patients had cardiovascular failure. More than 1 organ system failed in 3 enrolled patients. Among the entire cohort, evidence of infection was present in 5 patients, all of whom had surgery. Ten patients were transferred to the intensive care unit (ICU). The median duration of ICU stay was 15 days (range: 2–19 days), and the median duration of hospital stay was 9 days (range: 2–43 days). Two patients died in the hospital.

### 3.2 ROC analyses of scoring systems for predicting organ failure

**Figure 2:**
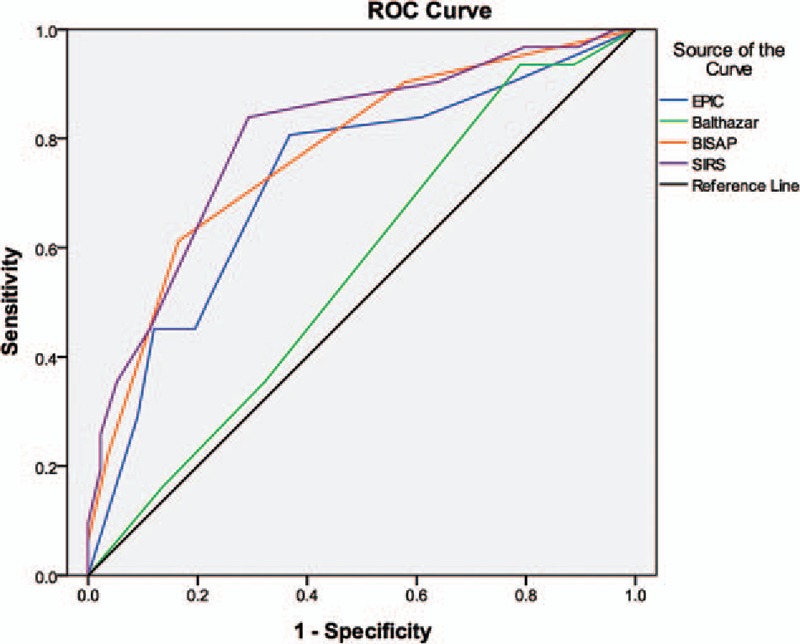
ROC curves of scoring systems for predicting organ failure in AP. The ROC curves show the AUC for the EPIC, Balthazar, BISAP, and SIRS scoring systems. AP = acute pancreatitis, BISAP = bedside index of severity in acute pancreatitis, EPIC = extrapancreatic inflammation on computed tomography, ROC = receiver operator characteristic, SIRS = systemic inflammatory response syndrome.

**Figure 3:**
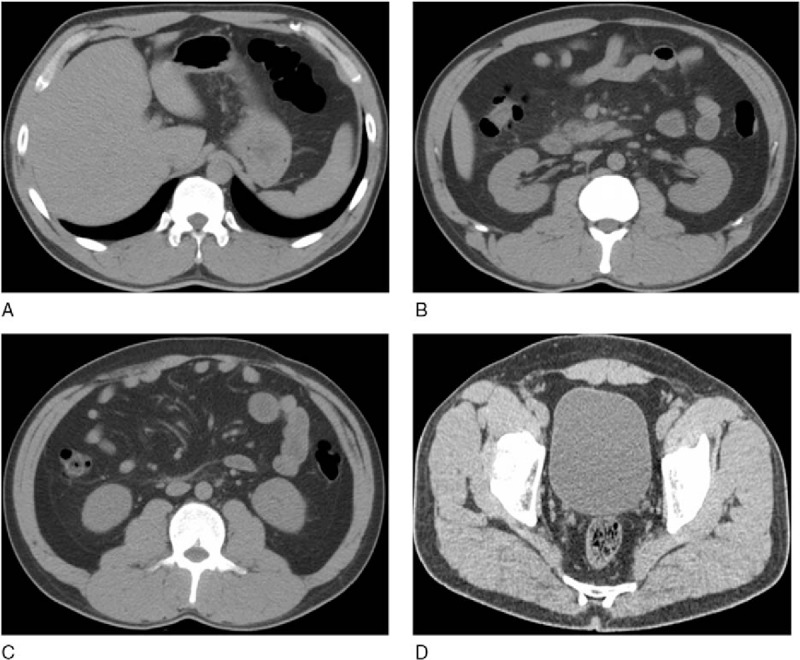
Transverse unenhanced abdominal CT scans of a 41-year-old man with AP who was discharged from the hospital 6 days after admission and had an EPIC score of 1. (A) Pleural effusions not present. (B) Right retroperitoneal inflammation. (C and D) Mesenteric inflammation and pelvic ascites not present. AP = acute pancreatitis, CT = computed tomography, EPIC = extrapancreatic inflammation on computed tomography.

**Figure 4:**
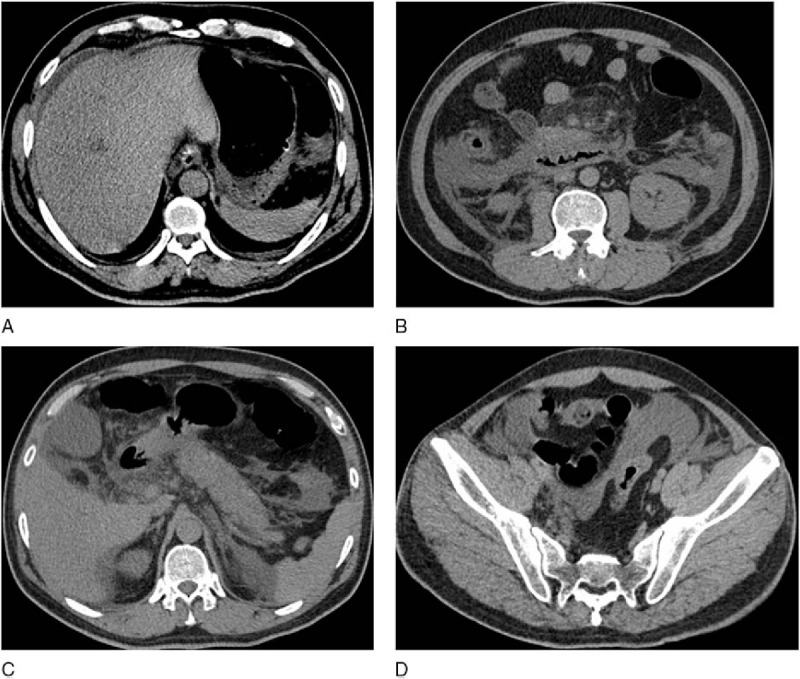
Transverse unenhanced abdominal CT scans of a 51-year-old man with AP who was discharged from the hospital 48 days after admission and had an EPIC score of 6. (A) Unilateral pleural effusions. (B) Bilateral retroperitoneal inflammation and mesenteric inflammation. (C and D) Ascites in perisplenic, perihepatic, interloop, and pelvic locations. AP = acute pancreatitis, EPIC = extrapancreatic inflammation on computed tomography.

ROC curves of the 4 scoring systems for predicting organ failure in AP are depicted in Fig. 2. The *P* value, AUC, cutoff value used to classify patients into high- and low-risk groups, and the sensitivity and specificity of all variables for predicting organ failure are shown in Table 4. The mean EPIC, Balthazar, BISAP, and SIRS scores were 2.7 ± 2.3, 2.2 ± 1.1, 1.0 ± 1.0, and 5.1 ± 2.8, respectively. The median EPIC score was 2 (range: 0–7). The AUCs of the EPIC and Balthazar scores were 0.724 (*P* = .000, 95% CI, 0.621–0.827) and 0.561 (*P* = .293, 95% CI, 0.455–0.666), respectively. An EPIC score cutoff of 3 or greater had a sensitivity and specificity for predicting organ failure of 80.65% (95% CI, 62.5–92.5) and 63.16% (95% CI, 54.4–71.4), respectively. AUCs of the BISAP and SIRS scores were 0.773 (*P* = .000, 95% CI, 0.679–0.867) and 0.801 (*P* = .000, 95% CI, 0.712–0.891), respectively. The EPIC score exhibited a trend toward lower accuracy compared to the SIRS score and the BISAP score in predicting organ failure, but these differences were not significant (EPIC vs. SIRS, *P* = .105; EPIC vs. BISAP, *P* = .251). Representative CT scans from a patient with a low EPIC score and a patient with a high EPIC score are shown in Figs. 3 and 4, respectively.

**Table 4:**
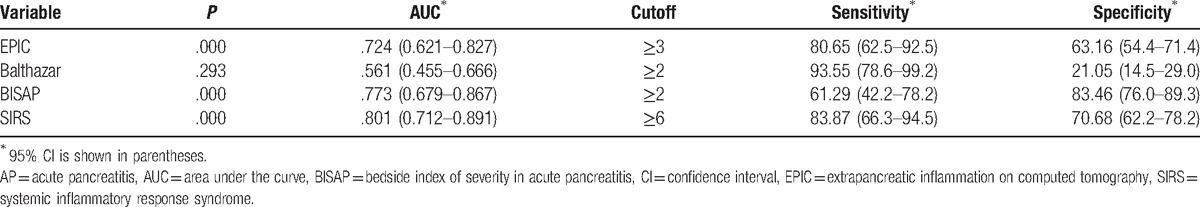
Performance statistics of variables for predicting organ failure in AP.

### 3.3 EPIC score for predicting severity of organ failure

The severity of organ failure was classified into 3 grades: grade 1 (no organ failure; EPIC score 0–2), grade 2 (transient organ failure; EPIC score 3–4), and grade 3 (persistent organ failure; EPIC score 5–7). A McNemar–Bowker value of 41.41 was obtained (df = 3, *P* < .001), with a Kappa value of 0.241 (*P* < .001). EPIC scores and Atlanta Marshall multiple organ dysfunction scores of AP severity differed significantly (*P* < .001). Kappa analysis indicated that EPIC scores had only a weak correlation with severity of organ failure. Spearman correlation analysis revealed a moderate correlation of EPIC score with severity of organ failure (*r* = 0.321, *P* < .001).

### 3.4 EPIC score for predicting number of failed organs

The number of failed organs was classified into 3 grades: grade 1 (no failed organs; EPIC score 0–2), grade 2 (1 failed organ; EPIC score 3–4), and grade 3 (2 failed organs; EPIC score 5–7). A McNemar–Bowker value of 47.966 was obtained (df = 3, *P* < .001). Kappa analysis indicated that EPIC score correlated weakly with the number of failed organs (Kappa = 0.214, *P* < .001). Spearman correlation analysis revealed a moderate correlation of EPIC scores with the number of failed organs (*r* = 0.343, *P* < .01).

## 4 Discussion

In this study, we used the 2012 revised Atlanta classification and found that the EPIC score, assessed by the presence of ascites, pleural effusion, and retroperitoneal edema, can be used to predict the occurrence of organ failure in the early phase of AP with an accuracy similar to those of the SIRS and BISAP scores and higher than that of the Balthazar score. We found that the EPIC score correlated moderately with the severity of organ failure. However, our results showed no significant difference in EPIC score between patients with transient organ failure and with persistent organ failure. This finding indicates that the EPIC score cannot be used to differentiate the severity of organ failure. Our results further revealed that the EPIC score correlated moderately with the number of failed organs, but we found no significant difference in EPIC score between patients with 1 failed organ and with 2 failed organs. This finding indicates that the EPIC score cannot be used to differentiate the number of failed organs.

The presence of extrapancreatic inflammation was reported to be an essential determining factor of AP severity in a previous study.^[24,25]^ More investigations^[26,27]^ found EPIC had a high accuracy for the early prediction of organ failure in patients with AP. Furthermore, EPIC scores can predict pancreatic pseudocysts in the early phase of severe AP.^[28]^ Conversely, distinct from our findings, Mortele and colleagues^[29]^ did not find a significant correlation between the prevalence of renal/perirenal involvement in complications and severity of pancreatitis.

However, few previous study has investigated the EPIC score correlated with the number of failed organs and the severity of organ failure. Therefore, to our knowledge, this study is the first time to study the relationship between EPIC score, number of failed organs and the severity of organ failure.

There are several patients with hypertriglyceridemia in our sample (n = 52), more than alcohol abuse. This is a bit different from the reference standard for AP. The patients were almost universally hypertriglyceridemia, it is possible influenced by the oily diet structure, or AP itself could lead to a hypertriglyceridemia. Yet, there were well-conducted studies suggesting that hypertriglyceridemia (HTG) AP was associated with a higher severity and complication rate.^[30]^ While Lindkvist and colleagues^[31]^ found that triglycerides may be a more important risk factor for acute pancreatitis than what has previously been estimated.

Our study has several limitations. First, it was a nonrandomized retrospective study with a medium-sized sample. Second, we analyzed only the subgroup of AP patients in the consecutive patient cohort who underwent CT within 24 hours after the onset of symptoms and laboratory examination at the first and third days of admission. Some patients with severe acute pancreatitis were discharged without medical advice, which may have contributed to the absence of hospital mortality in our cohort. Only 208 out of 325 patients (64.0%) were included in our study. This large number of exclusions may have introduced some selection bias, which might explain the low incidence of patients (only 3) with multiple organ failure. Third, we did not evaluate the interobserver variability in the EPIC score; however, the definitive EPIC score was determined by consensus in cases where the CT imaging findings between the 2 radiologists were inconsistent.

In conclusion, the EPIC score can be used to predict the occurrence of organ failure in the early phase of AP with similar to higher accuracy compared to conventional scoring systems. The EPIC score may be useful in predicting the duration of hospital stay for AP patients. However, it cannot be used to differentiate the severity of organ failure and number of failed organs.
